# The Role of Vitamin D Level and Related Single Nucleotide Polymorphisms in Crohn’s Disease

**DOI:** 10.3390/nu5103898

**Published:** 2013-09-27

**Authors:** Andre Y. O. M. Carvalho, Karen S. Bishop, Dug Yeo Han, Stephanie Ellett, Amalini Jesuthasan, Wen J. Lam, Lynnette R. Ferguson

**Affiliations:** 1 Presbyterian University Mackenzie, R. da Consolacao 930, Sao Paulo 01302-907, Brazil; E-Mail: 30952735@mackenzista.com.br or a.osava@auckland.ac.nz; 2 Discipline of Nutrition, FM&HS, University of Auckland, Private Bag 92019, Auckland 1142, New Zealand; E-Mails: dy.han@auckland.ac.nz (D.Y.H); s.ellett@auckland.ac.nz (S.E.); a.jesuthasan@auckland.ac.nz (A.J.); mao20_02@hotmail.com (W.J.L.); 3 Auckland Cancer Society Research Center, FM&HS, University of Auckland, Private Bag 92019, Auckland 1142, New Zealand; E-Mail: k.bishop@auckland.ac.nz; 4 Nutrigenomics New Zealand, University of Auckland, Private Bag 92019, Auckland 1142, New Zealand

**Keywords:** vitamin D levels, Crohn’s disease, *VDR*, *SCUBE3*, *PPP6R3*, *PHF-11*, SNPs, lifestyle

## Abstract

New Zealand has one of the highest rates of Crohn’s Disease (CD) in the world, and there is much speculation as to why this might be. A high risk of CD has been associated with deficient or insufficient levels of Vitamin D (Vit D), lifestyle as well as various genetic polymorphisms. In this study we sought to analyse the relevance of serum Vit D levels, lifestyle and genotype to CD status. Serum samples were analysed for 25-OH-Vitamin D levels. DNA was isolated from blood and cheek-swabs, and Sequenom and ImmunoChip techniques were used for genotyping. Serum Vit D levels were significantly lower in CD patients (mean = 49.5 mg/L) than those found in controls (mean = 58.9 mg/L, *p* = 4.74 × 10^−6^). A total of seven single nucleotide polymorphisms were examined for effects on serum Vit D levels, with adjustment for confounding variables. Two variants: rs731236[A] (*VDR*) and rs732594[A] (*SCUBE3*) showed a significant association with serum Vit D levels in CD patients. Four variants: rs7975232[A] (*VDR*), rs732594[A] (*SCUBE3*), and rs2980[T] and rs2981[A] (*PHF-11*) showed a significant association with serum Vit D levels in the control group. This study demonstrates a significant interaction between Vit D levels and CD susceptibility, as well as a significant association between Vit D levels and genotype.

## 1. Introduction

Both genetic and environmental factors have been associated with colonic disease, as well as cancers and other non-communicable diseases. Crohn’s disease (CD) is a form of Inflammatory Bowel Disease (IBD) that is chronic, relapsing, and causes inflammation of the intestine. CD is an autoimmune disease characterised by an inappropriate immune response and diminished tolerance towards indigenous intraluminal bacteria and food antigens, followed by a chronic inflammatory response [1,2,3,4]. Environmental and lifestyle factors such as smoking status, diet, intestinal microbiota, exposure to antibiotics, gender and age may contribute to the development of CD [2,5,6]. Both the hygiene hypothesis and Vitamin D (Vit D) hypothesis have been suggested as possible explanations for the increasing incidence and distribution of IBD [7,8,9]. However, it is not clear whether Vit D and genotype interact to enhance or diminish the risk of IBD development.

Vit D is a hormone that plays a role in both the innate and adaptive immune system, in addition to contributing to bone-formation and -density. Vit D can be obtained through the non-classical formation pathway via the consumption of oily fish, dairy, eggs, various cereals and other foods; by taking Vit D supplements; and approximately 90% is obtained through the classical pathway by the exposure of the skin to sunlight [10,11,12]. Due to initiatives in New Zealand (and elsewhere in the world) to protect against the harmful effects of overexposure to the sun’s UV rays, and a general deterioration in the nutritional content of the Western diet, population levels of Vit D have decreased over time [1,13].

Many non-communicable (NC) diseases, such as cardiovascular disease, immune disorders, obesity and common cancers are correlated with low Vit D exposure [14,15,16]. There is evidence that many NC diseases are, at least partly, preventable. For example, research supports the view that Vit D levels (metabolism and storage) influence and are influenced by obesity, such that per one kg/m^2^ increase in BMI leads to a decrease of 1.15% in 25(OH)D levels [16]. Thus, weight reduction may beneficially influence Vit D status.

In addition to lifestyle, genotype contributes to the development of CD, particularly via the presence of specific single nucleotide polymorphisms (SNPs) in susceptibility genes. The SNPs analysed in this study included those found in the *VDR* (*vitamin D receptor*), *SCUBE3* (*signal peptide, CUB domain, EGF-like 3*), *PPP6R3* (*Protein Phosphatase 6, Regulatory Subunit 3*) and *PHF-11* (*PHD finger protein-11*) genes. *VDR* is expressed in most cell types and, together with Vit D, has been shown to be an important regulator of the immune system.

SNPs located in the *VDR* gene, which have previously been associated with the risk of CD, were analysed. Additional genes, namely *SCUBE3,* *PPP6R3 and* *PHF-11*, were selected based on an analysis of the *VDR* pathway (unpublished data). *SCUBE3* appears to be associated with hypermethylation in some cancers [17,18], and is associated with the *VDR* pathway. The main functions of *PPP6R3* include transcription, translation, morphogenesis and cell-cycle regulation [19] and *PPP6R3* is also believed to be active in the *VDR* pathway. The fourth set of SNPs analysed were those found on the gene related to the formation of PHF-11. *PHF-11* has been associated with immune and inflammatory pathways [20] linked with chronic diseases. Related research has focused on the association with immune deregulation and chronic inflammation in asthma [21,22]. However, more recent studies have associated *PHF-11* activity with serum Vit D levels [23,24].

Although CD is an idiopathic disease, genetically predisposed individuals are likely to be more at risk of developing CD, given the required environmental conditions. These environmental conditions may include Vit D availability, smoking, infections, gut microbiota, amongst others [9,25]. The aim of this study was to investigate the association between serum Vit D levels and CD, and to determine the interaction between CD and genotype on Vit D levels, as well as to explore the interaction with age, gender and smoking status.

## 2. Experimental Section

A prospective case-control, population-based study was performed by the Nutrigenomics New Zealand programme to explore the association between serum Vit D levels and CD status, and to determine if there was an interaction with genotype.

### 2.1. Study Subjects

Study subjects were recruited between 2005 and 2013 by the Discipline of Nutrition in the Faculty of Medical and Health Sciences, and the Auckland Cancer Society Research Centre, The University of Auckland. Inclusion criteria for this study were: self-reported as Caucasian and in addition, for CD patients, to have CD status confirmed through clinical records. The exclusion criteria for the control group included IBD and a diagnosis of cancer (other than skin cancer). Cases and controls were not age or gender matched, and statistical adjustments were made where necessary. Ethnic groups other than Caucasian were excluded from analysis as their numbers were too small for the study to be adequately powered. In total, 633 patients (325 control and 308 CD patients) were recruited for this study. Cheek swabs and/or blood samples were collected. A total of 608 samples were available for serum Vit D testing (306 control and 302 CD patients). The study was conducted under ethical protocol MEC/04/12/011, authorised through the New Zealand Multi-Region Human Ethics Committee. All study participants gave written informed consent.

### 2.2. Vitamin D Determination

Vit D levels were measured in serum samples with a pre-coated 25-dihydroxy (OH) Xpress ELISA Kit (Immundiagnostik AG^®^, Bensheim, Germany). The assay provided a quantitative determination of serum 25-OH-Vitamin D by first using a releasing reagent to release 25(OH)-vitamin D from the 25(OH)-vitamin D-DBP-complex. A fixed amount of 25(OH)-vitamin D bound to the microtiter plate competed with 25(OH)-vitamin D in the serum sample for the binding of the antibody. The intensity of the colour reaction was inversely proportional to the amount of Vit D in the sample. The technician was blinded with respect to patient group affiliation to prevent any trending when processing the samples.

Seasonal variation for serum Vit D levels were classified into two seasons. These were based on the New Zealand daylight savings scheme. Daylight saving with high sunlight exposure (HSE) was regarded as extending from October to March, and covered the longer sunlight hours of summer. The non-daylight saving time was regarded as extending from April to September, when exposure to sunlight is lower (LSE: low sunlight exposure).

### 2.3. Genotyping

For this part of the study, seven SNPs were selected from four different genes on four chromosomes. The selected SNPs were previously associated with Vit D and/or CD, or with the *VDR* pathway. The SNPs were tested by using one of two different assays: Sequenom and ImmunoChip as follows: The Sequenom genotyping platform (Sequenom^®^, San Diego, CA, USA) using MALDI-TOF mass spectroscopy and MassARRAY technology with an iPlex system was used to analyse the SNPs related to the *VDR* gene (rs7975232 and rs731236) located on chromosome 12, and the *PPP6R3* gene (rs7109294 and rs10896349) located on chromosome 11. An ImmunoChip was used to analyse the SNPs found on the *SCUBE3* gene (rs732594) located on chromosome 6, and on the *PHF-11* gene (rs2980 and rs2981) located on chromosome 13 [26].

### 2.4. Statistical Analysis

The outcome of interest, serum Vit D levels as compared between CD and control group, was tested. Serum Vit D levels were log-transformed due to a skewed distribution of data. The estimated serum Vit D levels were determined by utilizing the exponential (anti-log) function. The association between serum Vit D levels and CD was fitted to a generalised linear model with an adjustment for three variables (smoking behaviour, age, and seasonal variation to sunlight exposure). An analysis of the interaction between CD and sunlight exposure on serum Vit D levels was also carried out. An additive model was fitted for gene-CD interaction on serum Vit D levels. SAS (V9.2 SAS Institute, Cary, NC, USA) and R [27] was used for statistical analyses. This resulted in a *p*-value of 0.05 as being statistically significant.

## 3. Results

### 3.1. CD Status, Serum Vitamin D Levels and Multivariate Analysis

**Figure 1:**
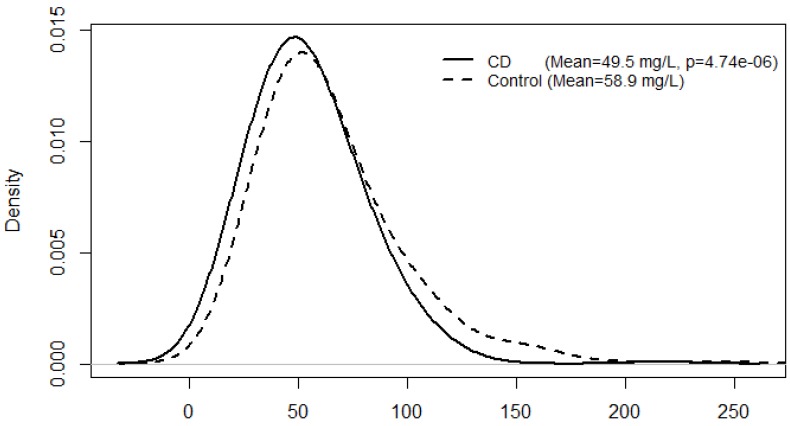
A density plot comparing serum vitamin D levels between Crohn’s Disease patients and controls.

A total of 608 serum samples (302 CD patients and 306 controls) were available for use in this study. Figure 1 shows the difference in serum Vit D levels between CD patients and controls before adjustment for variables (smoking behaviour, age, and seasonal variation of sunlight exposure). Serum Vit D levels were significantly lower in CD patients (mean = 49.5 mg/L) than those in controls (mean = 58.9 mg/L, *p* = 4.74 × 10^−6^). As shown in Table 1, serum Vit D levels remained significantly lower than those in the control group after adjustment for the variables (*p* = 2.97 × 10^−7^). Data was not presented on location of disease, extent and activity index, as it showed no significance with Vit D levels or any other variable tested. Table 2 shows a significant interaction between CD and seasonal variation of sunlight exposure. The highest serum Vit D levels were shown in the HSE-control group (61.0 mg/L) and the lowest were shown in the LSE-CD group (46.7 mg/L). From a multiple comparison among all pairs of means of high/low sunlight season and CD/control group, all pairs were significantly different, except LSE-control group with HSE-CD and LSE-control group with HSE-control.

**Table 1:** Multivariate analysis on serum vitamin D.

Variable	Status	Mean (95% CI)	*p*
**IBD**	CD (*n* = 302)	0.805 (0.741–0.874)	**2.97 × 10^−7^**
	Control (*n* = 306)	1.00	
**Smoking**	Yes (*n* = 144)	1.040 (0.946–1.144)	0.4165
	No (*n* = 464)	1.00	
**Seasonal variation of sunlight exposure**	HSE (*n* = 278)	1.138 (1.052–1.231)	**0.0013**
	LSE (*n* = 330)	1.00	
**Gender**	Female (*n* = 397)	0.998 (0.902–1.103)	0.9648
	Male (*n* = 211)	1.00	
**Age** (mean = 48.3, range of 10–91)		0.995 (0.992–0.997)	**4.19 × 10^−5^**

HSE = High Sun Exposure; LSE = Low Sun Exposure.

**Table 2:** Serum vitamin D by Crohn’s Disease status and seasonal variation of sunlight exposure.

Seasonal variation of sunlight exposure	CD status	*N*	Mean (95% CI)	*p*-values for multiple comparison	HSE-Control	LSE-CD	LSE-Control
HSE	CD	123	53.9 (49.7–58.5)	HSE-CD	0.0253	0.0079	0.3507
HSE	Control	155	61.0 (56.8–65.6)	HSE-Control		1.54 × 10^−7^	0.1695
LSE	CD	179	46.7 (43.6–49.9)	LSE-CD			1.31 × 10^−4^
LSE	Control	151	56.8 (52.8–61.1)	LSE-Control			

HSE = High Sun Exposure; LSE = Low Sun Exposure.

### 3.2. Gene-CD Interaction on Serum Vitamin D Levels

A total of seven polymorphisms were examined on serum Vit D levels with adjustment for confounding variables (Table 3). Two variants: rs731236[A] (*VDR*) and rs732594[A] (*SCUBE3*) showed a significant association with serum Vit D levels in CD patients. Four variants: rs7975232[A] (*VDR*), rs732594[A] (*SCUBE3*), and rs2980[T] and rs2981[A] (*PHF-11*) showed a significant association with serum Vit D levels in the control group.

**Table 3:** Gene-Crohn’s Disease interaction on vitamin D after adjustment for confounding variables *.

Gene	SNP effect		SNP × CD Interaction effect		
	SNP	Tested		Estimate	*p*
		Allele		(95% CI)	
*VDR*	rs7975232	A	CD	0.981 (0.904–1.066)	0.6535
			Control	1.101 (1.008–1.202)	0.0328
*VDR*	rs731236	A	CD	0.893 (0.825–0.966)	0.0050
			Control	1.058 (0.972–1.152)	0.1883
*SCUBE3*	rs732594	A	CD	0.917 (0.847–0.992)	0.0316
			Control	1.111 (1.007–1.226)	0.0363
*PHF-11*	rs2980	T	CD	1.023 (0.942–1.112)	0.5842
			Control	1.104 (1.012–1.205)	0.0265
*PHF-11*	rs2981	A	CD	1.024 (0.941–1.113)	0.5817
			Control	1.107 (1.014–1.209)	0.0229
*PPP6R3*	rs7109294	C	CD	1.023 (0.922–1.136)	0.6640
			Control	1.102 (0.976–1.243)	0.1152

* Confounding variables: smoking behaviour, age, and seasonal variation of sunlight exposure.

## 4. Discussion

### 4.1. Vitamin D Levels: Influence on Susceptibility to Crohn’s Disease

In this study, severe Vit D deficiency was regarded as 25-OH-Vit D levels <25 nmol/L and deficiency as levels between 26 nmol/L and 50 nmol/L [12] (1 ng/mL is equivalent to 2.5 nmol/L [11]). Relative insufficiency was classified as having serum Vit D levels between 51 and 72 nmol/L. Adequate or optimal levels were defined as being between 73 and 200 nmol/L [28]. Concentrations between 201 and 374 nmol/L are regarded as a breakpoint for toxicity [10,12].

This study demonstrates that there is a significant interaction between Vit D levels and CD susceptibility, with CD patients having lower levels of serum Vit D than control patients, during both HSE and LSE seasons (Table 2). During the LSE season, CD patients had a mean serum Vit D level that fell into the “deficient” category, whilst CD patients during the HSE season and control patients during both seasons showed mean insufficient levels. This indicates chronic Vit D insufficiency for both groups tested, with the CD patients having significantly lower serum Vit D levels than the controls. Vit D modulates the immune system and a large number of studies support the idea that low levels of Vit D are common amongst people with CD [29] and may lead to disease susceptibility [1,12,30,31], regardless of whether the CD is in remission [32].

Th1 and Th17 are pathogenic effector cells and regulation of these cells suppresses IBD [33]. All immune cells can express the *VDR*, but up-regulation of the *VDR* can only occur once the immune cells have been activated. 1,25 (OH) Vit D_3_ inhibits both Th1 and Th17 due to a direct effect on the production of IFNγ and IL-17, and an indirect effect on dendritic cells and macrophages [34]. Th-1 and 17 exacerbate the immune response in CD chronic inflammation [35,36], and it is clear that the *VDR* may suppress the CD associated inflammatory response. Cantorna *et al*. (2010) tested *VDR* deficient rats, demonstrating that *VDR* deficiency may lead to chronic, low-grade inflammation on the intestinal tract [37]. In the presence of *VDR* and Vit D deficiency or insufficiency, immune and inflammatory responses may lead to the pathogenesis of CD [38].

### 4.2. Age, Gender and Smoking Habits

An increase in age is related to an increase in immune and inflammatory deregulation, and greater susceptibility to disease. Therefore it is not surprising that elderly people show a higher frequency of auto-immune and inflammatory-related diseases [34,35,36]. In our study, aging and Vit D were shown to have a significant association (Table 1). This is expected as low levels of Vit D in older patients have been associated with cases of osteoporosis and bone health-related diseases [37,38]. However, we failed to show that gender was significantly related to Vit D levels (Table 1). We anticipated that female gender may be linked to lower Vit D levels as low levels have previously been associated with female gender [39], women receiving antenatal care [39], and osteoporotic postmenopausal women [38].

Smoking habit at diagnosis/enrolment was associated with CD status [39]. The current study reveals a trend towards smokers having higher Vit D levels, although this did not reach statistical significance (Table 1). In the literature, smoking has been largely associated with decreased Vit D levels [40,41], although one study reported higher Vit D levels amongst children exposed to cigarette-smoke [42]. It is possible that smokers have an increased exposure to sunlight relative to non-smokers, particularly since smoking has been banned in indoor public- and work-spaces in New Zealand. This could lead to higher Vit D levels amongst smokers during the summer months.

One of the limitations of this study includes the lack of data on the consumption of Vit D rich foods, Vit D supplementation, as well as use of tanning beds. All of these factors could increase Vit D levels. In future studies it would be wise to collect such information and test for association with serum Vit D levels before considering an interaction with genotype.

### 4.3. Single Nucleotide Polymorphisms Influencing Crohn’s Disease and Vitamin D Levels

The SNPs listed in Table 3 were tested for an interaction with Vit D. SNPs in the *VDR* (rs7975232 and rs731236), *SCUBE3* (rs732594) and *PHF-11* (rs2980 and rs2981) genes were found to be significantly associated with Vit D levels in either the CD or control groups, or both.

A VDR variant, rs731236[A], showed a significant association with serum Vit D levels in CD patients, whilst the VDR variant, rs7975232[A] showed a significant association with serum Vit D levels in the control group. Many of the polymorphisms found in the VDR gene (including rs7975232 and rs731236) do not change the amino acid sequence of the protein and are found in regulatory regions [43]. Despite this, such polymorphisms may regulate mRNA stability and thus influence gene expression [44]. This may account for the variation in association between a particular SNP and interaction between Vit D and CD/controls, and may be worth investigating further.

The significant association between SNPs in *PHF-11* and serum Vit D level found in this study amongst the control group, has been reported by others [23,24]. The *PHF-11* SNPs (rs2980 and rs2981) were previously reported as being related to chronic asthma [20]. It is generally accepted that low Vit D levels can be a risk factor for asthma [23,24] and therefore it was not surprising to find that these SNPs were associated with low serum Vit D levels in this study. However, these SNPs were not associated with CD, despite the fact that mutations in *PHF-11* are likely to increase the risk of CD due to the action of *PHF-11* on mediators of inflammatory and immune responses [20,21,30].

A SNP (rs732594) in the *SCUBE3* gene was also associated with both Vit D levels and with CD. Very little literature exists on polymorphisms in *SCUBE3* but this gene appears to be associated with hypermethylation in some cancers [17,18]. *SCUBE3* is also involved in angiogenesis and is strongly expressed in aggressive lung carcinomas [45,46]. *SCUBE3* is likely involved in immune response and therefore it is not unexpected that there is an association with low Vit D levels as well as CD, as both of these conditions are associated with deregulation of the immune system.

SNPs tested in *PPP6R3* were not found to be associated with Vit D levels. The reason these SNPs were analysed was due to the fact that *PPP6R3* was thought to be involved in the *VDR* pathway when we used pathway analysis software (GATHER) to analyse Immunochip data for the detection of pathway associations. Very little literature exists on *PPP6R3* in general, and no association with Vit D or CD appears to have been reported. However, a break point has been reported in the *PPP6R3* gene when analysing samples from primary myeloma patients [47]. It may be of interest to investigate the presence of this *PPP6R3* break point in CD patients in the future.

## 5. Conclusions

It is clear that people with CD have significantly lower Vit D levels than a normal, healthy population, and that these levels vary according to age. In both cases and controls Vit D levels were associated with season, with higher Vit D levels found during the longer daylight months. Numerous experimental studies support the modulation of the immune system via Vit D in the pathology of CD, and a number of SNPs were found to be associated with lower Vit D levels in both the CD and/or control groups. The SNP rs732594, found in *SCUBE3*, was found to be associated with low Vit D levels in both CD and healthy people. Future studies are expected to focus on elucidating the mechanism by which Vit D modulates the immune response in CD; how genotype might determine response and how this knowledge might be used to enhance treatment; as well as benefits that might be derived from various levels and duration of Vit D supplementation.
